# Changes in Surgical Volume in Military Medical Treatment Facilities and Military Surgeon Clinical Combat Readiness During the COVID-19 Pandemic

**DOI:** 10.1097/AS9.0000000000000308

**Published:** 2023-06-21

**Authors:** Andrew J. Schoenfeld, Hoa T. Ho, Roman J. Schoenfeld, Christian Coles, Tracey P. Koehlmoos

**Affiliations:** From the * Center for Surgery and Public Health, Department of Orthopaedic Surgery, Brigham and Women’s Hospital, Harvard Medical School, Boston, MA; † Department of Preventive Medicine and Biostatistics, Uniformed Services University of the Health Sciences, Bethesda, MD; ‡ Medfield Public Schools, Medfield, MA; § Department of Preventive Medicine and Biostatistics, Uniformed Services University of the Health Sciences, Bethesda, MD; ‖ Department of Preventive Medicine and Biostatistics, Uniformed Services University of the Health Sciences, Bethesda, MD.

## Abstract

Mini-abstract

In this retrospective study, we evaluated changes in measures of surgeon clinical combat readiness within the military health system during the COVID-19 pandemic. We found a 36% reduction in surgical knowledge and skills as compared to pre-COVID. Sizable reductions were encountered for surgery for colectomy (−50%) and aneurysm repair (−61%).

## INTRODUCTION

The COVID-19 pandemic disrupted the US healthcare system in an unprecedented manner, including the reallocation of resources for the management of patients with COVID along with the suspension of the performance of elective and urgent surgical interventions. Within the military health system (MHS), personnel was also deployed to the civilian setting to provide material aid in overwhelmed sectors. The MHS has a unique nature, with federal (direct care) and private sector environments. Active-duty personnel, retirees, and dependents receive care through 1 of the 2 environments, based on location and provider availability.^1^ This mechanism allowed for effective “load shedding” of surgical backlogs and the efficient transfer of cases from military treatment facilities to civilian hospitals during the COVID-19 pandemic, which minimized any adverse impact on surgical care delivery within the MHS overall.^2^ However, these same strategies could result in reduced clinical volume among military treatment facilities, with deleterious effects on the expeditionary readiness of surgical teams within the armed forces.^3,4^ Concerning reductions across the MHS in surgical volume associated with clinical readiness were already identified before the onset of COVID-19.^4^ These stemmed, in part, from changes in procedural numbers in the direct care setting due to the introduction of a new electronic medical record system, shedding of Medicare-eligible retirees due to reduced primary care capacity and personnel availability.^4–6^ Such issues within the direct care environment persisted during the period of COVID-19, and the adverse effects on surgical volume could have been exacerbated as a result. In this context, we sought to evaluate changes in objective measures of surgeon clinical combat readiness within the MHS during the COVID-19 pandemic.

## METHODS

A query of the MHS Data Repository^1^ was performed for individuals receiving surgery for a battery of urgent and elective conditions, within direct or private sector care, between March and September 2017 to 2020 among beneficiaries aged 18 to 64 years. The design of the MHS Data Repository allows for the capture of all procedures performed across all military treatment facilities in the MHS, and care delivered through the private sector to MHS beneficiaries. Data collection was restricted to March to September in each year to mirror the time window of the COVID pandemic in the fiscal year 2020 (eg, March–September 2020). The procedures selected are representative of general surgery and orthopedics and have been used to study surgical health policy within the MHS and other healthcare settings in the past.^2^ The period 2017 to 2019 was considered prepandemic, and 2020 was considered the time of COVID-19.^1^ Using previously published methodology,^4,7^ we assigned knowledge, skills, and abilities (KSA) scores for each surgical procedure that was performed in direct care or the private sector. The KSA metric was developed by the US Armed Forces to quantify clinical readiness for military medical deployment.^7^ Values were averaged over the course of the pre-COVID time period to allow for accurate comparisons to the time of COVID. Differences in the change in KSA within direct care and private care were assessed between the pre-COVID and COVID timeframes for all surgical procedures using the *χ*^2^ test. Significance was set at *P* < 0.05.

## RESULTS

In the time period before COVID, among the procedures we considered, there were an average of 10,690 cases performed in the military setting, totaling 601,456 KSA points. This was significantly reduced following COVID to 6,599 procedures and 387,055 KSA points, which represented a 36% reduction as compared to pre-COVID levels (*P* < 0.001). Decreases in procedures outsourced to the private sector (13,768 vs 11,447), and resultant lost KSA points (709,667 vs 606.079; 15% change) were also appreciated. However, this reduction was less than that realized for military facilities. Among high-volume urgent KSA procedures, significantly different reductions were appreciated in direct care compared to the private sector (Table 1). These included appendectomy (−31% in direct care vs −11% in private sector), cholecystectomy (−37% in direct care vs −15% in private sector), and abdominal aortic aneurysm repair (−61% in direct care vs −33% in private sector). Similarly significant findings were encountered among elective procedures (Table 2), including inguinal hernia repair (−33% in direct care vs −17% in private sector) and bariatric surgery (−42% in direct care vs −15% in private sector).

**TABLE 1.** The List of Urgent Procedures and KSA Values Performed in Direct Care and Private Sector Care During the Period of the COVID-19 Pandemic and the Prepandemic Period

Procedure	Direct Care Procedures (Pre-COVID)	Total Direct Care KSA Value (Pre-COVID)	Private Sector Care Procedures (Pre-COVID)	Total Private Sector Care KSA Value (Pre-COVID)	Direct Care Procedures (COVID)	Total Direct Care KSA Value (COVID)	Private Sector Care Procedures (COVID)	Total Private Sector Care KSA Value (COVID)	Change in Direct Care	Change in Private Sector Care	*P* value
Appendectomy	1,295	188,997	1,390	202,940	893	130,378	1,240	181,040	−31%	−11%	<0.001
Cholecystectomy	2,092	238,488	2,459	280,269	1,326	151,164	2,101	239,514	−37%	−15%	<0.001
Mastectomy	169	16,393	202	18,546	108	10,476	157	15,229	−36%	−22%	<0.001
Surgery for cervical myelopathy	306	3,666	420	5,034	162	1,944	331	3,972	−47%	−21%	<0.001
Surgery for spinal metastatic disease	4	42	2	18	1	12	3	36	−71%	100%	<0.001
Colectomy for diagnosis of cancer	32	4,672	44	6,424	16	2,336	31	4,526	−50%	−30%	<0.001
CABG	112	5,464	168	8,208	73	3,577	136	6,664	−35%	−19%	<0.001
AAA repair/reconstruction	18	2,826	23	3,533	7	1,099	15	2,355	−61%	−33%	<0.001
Total	4,026	460,548	4,705	525,971	2,586	300,986	4,014	453,336	−35%	−14%	<0.001

AAA indicates abdominal aortic aneurysm; CABG, Coronary Artery Bypass Grafting.

**TABLE 2.** The List of Elective Procedures and KSA Values Performed in Direct Care and Private Sector Care During the Period of the COVID-19 Pandemic and the Prepandemic Period.

Procedure	Direct Care Procedures (Pre-COVID)	Total Direct Care KSA Value (Pre-COVID)	Private Sector Care Procedures (Pre-COVID)	Total Private Sector Care KSA Value (Pre-COVID)	Direct Care Procedures (COVID)	Total Direct Care KSA Value (COVID)	Private Sector Care Procedures (COVID)	Total Private Sector Care KSA Value (COVID)	Change in Direct Care	Change in Private Sector Care	*P* value
Rotator cuff repair	1,037	16,621	1,420	24,140	639	10,863	1,206	20,502	−38%	−15%	<0.001
Discectomy	471	5,652	660	7,914	298	3,576	513	6,156	−37%	−22%	<0.001
Bariatric surgery	602	39,130	728	47,288	349	22,685	616	40,040	−42%	−15%	<0.001
Cataract surgery	2,658	13,290	3,667	18,335	1,622	8,110	2,966	14,830	−39%	−19%	<0.001
Inguinal hernia repair	660	44,187	840	56,280	441	29,547	699	46,833	−33%	−17%	<0.001
Total knee arthroplasty	755	12,827	1,091	18,547	411	6,987	881	14,977	−46%	−19%	<0.001
Total hip arthroplasty	483	8,203	657	11,169	253	4,301	549	9,333	−48%	−16%	<0.001
TURP for diagnosis other than cancer	0	0	1	42	0	0	3	72	0%	200%	N/A
Total	6,664	140,908	9,063	183,697	4013	86,069	7,433	152,743	−39%	−17%	<0.001

TURP indicates Transurethral Resection of the Prostate.

## DISCUSSION

In this study, we found a sizable reduction in KSA points available to direct care providers in 2020, including 36% fewer points when compared to the prepandemic timeframe. Prior work, antedating COVID-19, had already highlighted a 26% decrease in surgical procedures generating KSAs between 2015 and 2019.^4^ Although declinations in the civilian sector were also appreciated in our work, these were about two times fewer as compared to military facilities. The performance of the private sector in this investigation is aligned with other studies focusing on the civilian health sector in general. Such studies have indicated that after initial reductions following the onset of COVID, surgical volumes rebounded to approximate prepandemic levels by the end of 2020, or 2021 at the latest.^8–10^

Limitations to this investigation include its retrospective design, restricted surveillance window, particularly with respect to the period of the COVID-19 pandemic, and limited granularity regarding the reasons cases were performed in the direct or purchased care sectors. We also acknowledge that issues may be present in case identification due to differences in the use of the surgical coding algorithms we employed here. In addition, we recognize that some military surgeons do elect to engage in off-duty employment that may contribute to their surgical case volume, or participate in military-civilian partnerships and these metrics would not be captured in our determinations of KSA scores. Some may contend that the military affiliation of the population covered by the MHS is also a limitation, but numerous studies have previously demonstrated that MHS beneficiaries are representative of the US demographic aged 18 to 64 years in terms of socio-ethnic, economic, vocational, and occupational characteristics.^1,2^

Our findings are cause for concern regarding the expeditionary readiness of surgical teams within the armed forces and may adversely impact military surgeon job satisfaction and retention.^3,4^ From a policy perspective, a near-term priority would include maximizing the recapture of surgical cases currently being transferred to the private sector, especially high-value KSA procedures including appendectomy, cholecystectomy, colectomy, and aneurysm repair. In the post-COVID environment, increasing civilian-military clinical partnerships and expanding federal beneficiaries eligible for care at military facilities could also improve the surgical volume and consequent expeditionary readiness.

## ACKNOWLEDGMENTS

A.S., H.T.H., and T.P.K: had full access to all the data in the study and take responsibility for the integrity of the data and the accuracy of the data analysis and study concept and design. A.S., H.T.H., R.J.S., and T.P.K: acquisition of data. A.S., H.T.H., R.J.S., C.C., and T.P.K: analysis and interpretation of data, drafting of the article, and administrative, technical, or material support. A.S., C.C., and T.P.K.: critical revision of the article for important intellectual content. A.S., H.T.H., and R.J.S: statistical analysis. A.S., T.P.K.: obtained funding and study supervision.
